# Outcomes of telephone-delivered low-intensity cognitive behaviour therapy (LiCBT) to community dwelling Australians with a recent hospital admission due to depression or anxiety: MindStep™

**DOI:** 10.1186/s12888-018-1987-1

**Published:** 2019-01-03

**Authors:** Sharon Lawn, Nancy Huang, Sara Zabeen, David Smith, Malcolm Battersby, Paula Redpath, Fiona Glover, Anthony Venning, Jane Cameron, Kate Fairweather-Schmidt

**Affiliations:** 1 Flinders Human Behaviour and Health Research Unit, College of Medicine and Public Health, Flinders University, PO Box 2100, Adelaide, SA 5001 Australia; 2 Remedy Healthcare Group, 390 St Kilda Rd, Melbourne, VIC 3004 Australia; 3 Mental Health Services – Southern Adelaide Local Health Network, College of Medicine and Public Health, Flinders University, PO Box 2100, Adelaide, SA 5001 Australia

**Keywords:** Low-intensity cognitive behaviour therapy, Community mental health service, Depression, Anxiety, Prevention and early intervention, Private health insurance, Hospital admission, Improving access to psychological therapies

## Abstract

**Background:**

In 2006, the British government launched ‘Improving Access to Psychological Therapies’ (IAPT), a low intensity cognitive behaviour therapy intervention (LiCBT) designed to manage people with symptoms of anxiety and depression in the community. The evidence of the effectiveness of IAPT has been demonstrated in multiple studies from the UK, USA, Australia and other countries. MindStep™ is the first adaptation of IAPT in Australia, delivered completely by telephone, targeting people with a recent history of a hospital admission for mental illnesses within the private health system. This paper reports on the outcome of the first 17 months of MindStep™ implemented across Australia from March 2016.

**Methods:**

This prospective observational study investigated the MindStep™ program in a cohort of clients with a recent hospitalisation for mental illnesses. The study used quantitative methods to compare pre-post treatment clinical measures (*N* = 680) using Patient Health Questionnaire (PHQ-9) and the Generalised Anxiety Disorder (GAD-7). This study also included in-depth interviews with participants (*N* = 14) and coaches (*N* = 4) to determine the feasibility and acceptability of the program.

**Results:**

Of the 867 clients referred to MindStep™, 757 had initial assessments by phone making an enrolment rate of 87.3%. Following assessment, 680 commenced treatment and of them, 427 (62.7%) completed treatment. According to ‘per-protocol’ analysis (*N* = 427), there was a large effect size for post-treatment PHQ-9 (d = 1.03) and GAD-7 (d = 0.99) scores; reliable recovery rate was 62% (95% CI: 57–68%). For intent-to-treat analysis using multiple imputation (*N* = 680), effect sizes were also large for pre-post treatment change: PHQ-9 (d = 0.78) and GAD-7 (d = 0.76). The reliable recovery rate was 49% (95% CI: 45–54%). Qualitative findings supported these claims where participants were positive about MindStep™ and found the telephone delivery and use of mental health coaches highly acceptable.

**Conclusions:**

MindStep™ has demonstrated encouraging outcomes that suggest LiCBT can be successfully delivered to people with a history of hospital admissions for anxiety and depressive disorders and achieve target recovery rates of > 50%. Other promising evaluation findings indicate the MindStep™ option is acceptable, feasible and safe within the stepped models of mental health care delivery in Australia.

**Electronic supplementary material:**

The online version of this article (10.1186/s12888-018-1987-1) contains supplementary material, which is available to authorized users.

## Background

In Australia, anxiety and depressive disorders are common, leading to significant burden on people’s lives and the community [1]. The latest National Survey of Mental Health and Wellbeing showed that around one million adults (6.2%) experienced a 12-month depressive disorder and over two million experienced a 12-month anxiety disorder (14.4%) in Australia (with a population of 16 million) [2]. The survey also reported that only 40% of this population accessed any healthcare services [2], despite an annual AUD 974 million spent on health care costs and another AUD 11.8 billion attributed to productivity loss [3]. However, for people with chronic and recurring symptoms, transitioning care from acute hospitals to the community can be fragmented and difficult to navigate [4]. Existing service models produce unintended barriers to accessing care [5, 6]. It is estimated that only one third of people diagnosed with depression actually receive any formal support [7], and up to 50% of these people may not be receiving evidence-based treatments [7, 8].

With traditional therapist-led models of care and ever-increasing demand, prevention and early intervention are often neglected within the continuum of care. Critics state that, “because of significant waiting lists those people who might make the greatest gains from Cognitive Behavioural Therapy (CBT) i.e. those with mild to moderate depression, anxiety and panic, are least likely to be referred…”([6], p., 676).

In 2006, the British government launched an innovative program called ‘Improving Access to Psychological Therapies’ (IAPT) to address this unmet demand for treatment of anxiety and depression in the UK population [9]. It was associated with the creation of a new workforce to deliver early and low intensity interventions consistent with the National Institute for Health and Care Excellence (NICE) guideline recommendations [10]. The potential effectiveness of this model has been shown in a number of cohort studies in the UK, USA, Australia and other countries [11–16], with recent literature specifying how it fits into stepped models of mental health care [5]. Findings from a more recent Norwegian study have further contributed to the emerging evidence-base where recovery rates are reported from both complete case and intent-to-treat perspectives [17]. This and other previous studies provide a conduit for establishing high quality evidence within the Australian context including public and private hospitals, with the goal to improve client outcomes upon discharge and future potential to reduce hospital readmissions.

### MindStep™

MindStep™^1^ is the latest adaptation of IAPT in Australia, which is an ‘opt-in’ service offered to people from participating private health insurance funds with a recent hospital admission due to depression or anxiety. This is the first application of IAPT in a private health insurance context, to address symptom recovery and subsequent hospital utilisation.

It is delivered over 6–8 weeks, telephonically by LiCBT coaches under close clinical supervision. The first clinical assessment identifies suitability for the intervention using a semi-structured clinical interview, including risk assessment. Subsequent sessions follow a structured guided LiCBT format between coach and client, using the person’s own problem statements, goals, planned actions and workbook exercises, supported by guided self-help, social prescribing to engage the person with their social networks and signposting to community services [11]. After initial assessment and up to six treatment sessions, clients are offered a 1-month, 3-month and 6-month follow-up to monitor whether improvements are maintained. Baseline symptom scores for *Patient Health Questionnaire* (PHQ-9), *Generalized Anxiety Disorder* (GAD-7) and Work and Social Adjustment Scale (WSAS) are obtained at each contact.

The average duration of an assessment is 1 h and duration of treatment sessions is approximately 30 min. The service uses a proprietary software system to collect client information, support case management and clinical supervision of mental health coaches. More information on the program can be found in the Additional file 1.

MindStep™ was launched in October 2015 and was fully functional from March 2016. This paper reports on the first 17 months of MindStep™ outcome, implemented across Australia starting from March 2016.

### Study aims

The study aimed to assess:

1. The feasibility and acceptability of MindStep™ in the Australian private health system; and
2. Whether MindStep™ can achieve benchmark recovery rates of > 50% in people with recent mental health hospital admission [18]

## Methods

### Study design

This prospective observational study investigated the MindStep™ program in a cohort of clients with a recent hospitalisation for mental illnesses. The study used quantitative methods to compare pre-post treatment clinical measures (*N* = 680) using Patient Health Questionnaire (PHQ-9) and the Generalised Anxiety Disorder (GAD-7). This study also included in-depth interviews with participants (*N* = 14) and coaches (*N* = 4) to determine the feasibility and acceptability of the program.

Data were collected to answer the following key questions:

1. What is the recovery, reliable improvement and reliable recovery rates of MindStep™? How do they compare with the UK IAPT benchmark?
2. What is the enrolment, completion, drop out and step up rates?
3. Is there an optimal number of sessions to maximise recovery rates?
4. Do clients with varying initial symptom severity experience any differences in their recovery rates? If so, what are the implications for future program selection criteria?
5. Are the recovery rates relatively consistent across coaches?
6. Were clients satisfied with MindStep™?

### Setting

Six private health funds referred their members from all over Australia to the program. However, the LiCBT coaches delivered the program from Melbourne MindStep™ office by telephone and the clinical supervisors were based at Flinders University, Adelaide.

### Participants and recruitment

Individuals were identified as eligible for the program following an acute hospital admission for anxiety and/or depression, confirmed by the member’s claims data and ICD-10 codes. Once identified, health funds offered individuals an opportunity to opt-out via email or telephone, before passing on their contact details to MindStep™. At least 5 attempts to contact the client were made by MindStep™ to enrol (with consent) and ensure there were no contraindications (such as imminent risk to self or others, active psychosis or chronic substance misuse). At this first contact, consent for the use of de-identified clinical data for the purpose of evaluation of the trial was sought from all client participants. At this time, a further opt-out option was offered to them which did not affect their receipt of the intervention; 680 clients provided consent.

### Quantitative data collection and analysis

#### Data sources and variables

With consent and ethics approval, de-identified data were extracted from the clinical software system from March 2016 to July 2017. This data included age, sex, socio-economic status, marital status, employment status, assigned coach, number of sessions attended and clinical measures (PHQ-9, GAD-7) to assess key outcomes of interest and to determine reasons of ‘end of care’ (Table 1) in answering the research questions.

**Table 1:** Outcomes of interest and definitions of ‘end of care’ categories for analysis

	Defining criteria
Measures	
Recovery	Number of clients who were at or above *caseness* (PHQ-9 > 9 and/or GAD-7 > 7) before treatment, then below *caseness* in both PHQ-9 and GAD-7 after treatment
Reliable improvement	Number of clients who demonstrated an improvement of ≥6 on PHQ-9 and/or ≥ 4 on GAD-7, regardless of whether this change meant that they were still in *caseness*. Reliable improvement refers to the improvement in the PHQ-9 and GAD-7 scores that is sufficient to conclude that the improvement in the scores is beyond that which could be attributed to measurement error. It is an important measure for showing improvement in clients with more severe baseline symptom scores
Reliable recovery	Number of clients that both moved to recovery and showed reliable improvement
Clinical software category	
Completed all sessions	Client has completed at least assessment plus 2 or more treatment sessions (up to a total of 6) and deactivated as completed.
Completed all sessions and stepped up to other services	Client completed up to 6 sessions plus assessment (and was considered as completed program) but also required to be stepped up to higher intensity mental health services.
Not suitable at assessment	Client has complex or multi-morbid mental health conditions and deemed unsuitable for MindStep™.
Client declined treatment	Client made an informed choice not to participate in MindStep™ after the first assessment or first treatment session.
Stepped up	The service did not meet the clients’ level of clinical need following assessment and before receiving a minimum of 2 further sessions, and was stepped up to higher intensity mental health services.
DNA	Client cancelled in advance of appointment or did not respond to contact attempts at the scheduled appointment.
Deceased	Client is deceased post referral.
Client drop out	Client drops out post-assessment before receiving a minimum of 2 further sessions; and unable to re-establish contact with no response from contact attempts.

Source: National Health Service 2011, UK [18]

### Statistical methods

#### Main analysis

The primary analysis was based on a ‘per-IAPT protocol’ to investigate effect size estimates, recovery rate and reliable recovery rate in primary outcomes PHQ-9 and GAD-7 at post-treatment. This approach sought to estimate treatment effect if all clients completed their therapy providing ‘missing at random’ (MAR; probability of missing data is dependent on observed values and possibly covariates but independent of unobserved values [19]) was a reasonable assumption for treatment non-adherers. Secondary analyses followed an intent-to-treat (ITT) principle where all clients were included in the analysis to avoid potential effects from therapy drop out [20]. Inferences from ITT analysis are generalizable to therapy effectiveness in everyday clinical practice but may underestimate effects in the presence of non- adherence to therapy protocol. We also performed a modified ITT (mITT) analysis to assess effect estimates when excluding clients who were stepped up to high intensity treatment. Statistical analyses were conducted using Stata 15.1 [21].

Two different approaches were used for ITT analyses to handle missing PHQ-9 and GAD-7 data. First, the single imputation method of last observation carried forward (LOCF). Second, multiple imputation (MI) by chained eqs. A main feature of chained equations is its ability to handle different variable types (e.g., continuous and binary) [22]. To reduce the chance of bias from missing data, multiple imputation by chained equations with 40 imputations was used [22]. Covariates included in our imputation models were baseline gender, age, SES, employment, PHQ-9, GAD-7, and WSAS. Post-treatment variables were PHQ-9 and GAD-7. The results obtained from 40 completed-data analyses were then combined into a single multiple-imputation result for effect size, recovery rate and reliable recovery rate.

As MI models were based on the assumption that data were at least missing at random (MAR), sensitivity analyses were performed via a pattern mixture approach with multiple imputation (MI). This was done separately for PHQ-9 and GAD-7 by (1) imputing the missing response values at post-treatment using chained equations for 40 imputations; (2) checking that these results were the same as for the linear regression for observed data only; and (3) for imputed data, increasing the mean response above that predicted under MAR, and re-analysed. If the data were missing not at random (MNAR; probability of missingness is dependent on both observed and unobserved values), it was likely that the mean at post-treatment (for clients for whom it was missing) was higher than that predicted under MAR. Utilising the findings from LOCF method, the distributions *N*(M = 5.0, SD = 5.0) and *N*(4.0, 4.0) were used for PHQ-9 and GAD-7 respectively to summarise the assumed difference between MNAR and MAR. Variants of these distributions were also tested to reveal the effects of different levels of uncertainty.

Effect size estimate Cohen’s *d* was calculated using one-sample *t-*test for difference scores between pre- and post-treatment, assuming null hypothesis equal to zero. The reason for this approach was to account for dependency or correlation within individual assessment scores. The *t-*value was directly used in the formula: *d* = *t*/$\sqrt{N}$ [23]. The 95% confidence intervals were calculated by finding the non-centrality parameters *λ*_*lower*_ and *λ*_*upper*_ using Stata function *npnt(df, t, p)* [21] and then transformed back to the effect-size scale with ${d}_{lower/ upper}=\frac{\lambda_{lower/ upper}}{\sqrt{N}}$. For imputed MI data, Cohen’s *d* used adjusted means and standard errors for the variability between imputations in an immediate form (i.e., summary statistics entered as arguments) of a one-sample *t*-test.

#### Secondary exploratory analyses

We also used Chi-square and Fisher’s exact test (to account for low cell counts) [24]. Post-hoc Phi and Cramer’s V [24] were carried out to determine the association between symptom severity and outcomes and to measure heir effect sizes, using SPSS (v22.0) [25]. The same tests were used to assess any differences among coaches’ performance. Finally, General Linear Model (GLM) [26] was employed to check the differences among coaches in terms of number of treatment sessions.

#### Qualitative data collection

Interviews were conducted with MindStep™ coaches and clients (including people who dropped out). The aim was to recruit sufficient client participants to ensure the capture of views across the range of jurisdictions, and demographic and clinical features and outcomes from the intervention. The interviews were guided by a semi-structured questionnaire that was initially developed by the clinical supervisor and the qualitative research officer. The questionnaire was further refined by the team. The key questions were: participants’ experience of the program; confidence in managing mental health after the intervention; most and least helpful aspects of the program; how the program could be improved; and, any other comments. Client interviews took place via telephone (as most were outside of South Australia), whilst coach interviews were undertaken face-to-face. All interviews lasted between 30 and 60 min.

#### Qualitative data analyses

Interviews were recorded, transcribed and analysed by Flinders research team members using Framework Analysis [27], chosen to best answer the established research questions. The data was independently coded by the qualitative research officer with input and under the supervision of the clinical supervisor and the research leader who had expertise in qualitative methods. Together, they established tentative themes that were then presented to the remaining research team members for discussion to ensure rigor as part of the research team agreeing on and finalising the framework for presenting the final qualitative findings.

## Results

### Quantitative findings

#### Participants

**Fig. 1:**
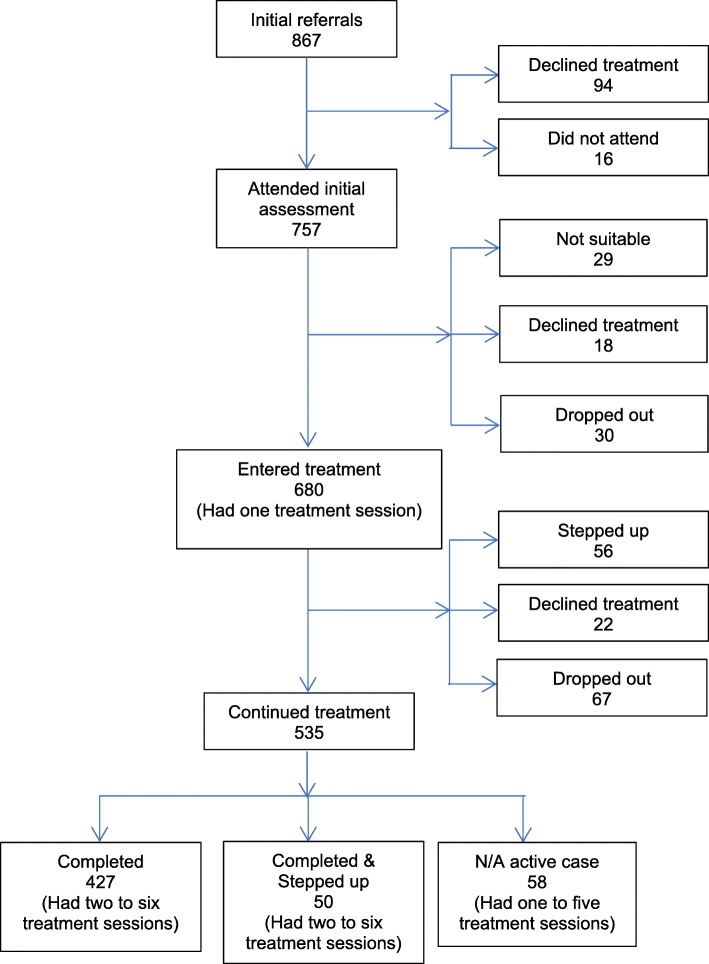
Clients - flow diagram

Between March 2016 and July 2017, 867 clients were referred to MindStep™.757 attended the initial telephone assessments making an enrolment rate of 87.3%. Of 757 clients, 680 commenced treatment (had at least one treatment session) with 535 clients continuing the program. ‘Reasons for end of care’ showed that of the remaining clients who attended the initial assessments (*N* = 222), 29 (3.8%) were not suitable at assessment, 56 (8.2%) were stepped up, 40 (5.3%) declined further treatment, and 97 (12.8%) dropped out (Fig. 1).

**Fig. 2:**
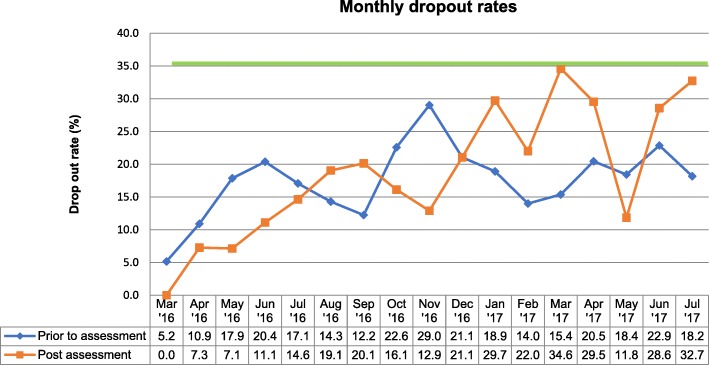
MindStep™ monthly dropout rate

Figure 2 also shows month-wise dropout rates before and after attending the assessment.

The overall program completion rate was 62.7% as 427 of 680 clients completed treatment (had minimum two treatment sessions). ‘Reasons for end of care’ also showed that 50 (7.3%) clients were stepped up after completing six treatment sessions, and 58 (8.5%) were active cases at the time of reporting.

#### Missing data

Although most baseline data was available there was missing data for employment (105/680, 15.4%), PHQ-9 and GAD-7 (5/680, 0.7%) and WSAS (10/680, 1.5%). Missing data rates at post-treatment for primary outcome PHQ-9 was 50/680 (7.4%) and GAD-7 54/680 (7.9%).

#### Sample characteristics

To analyse the key measures, 680 clients were considered who entered the treatment; Table 2 presents their demographics and clinical characteristics.

**Table 2:** Demographic and clinical characteristics of 680 clients who entered treatment

		Completers (*N* = 427)	Non-completers (*N* = 253)
Demographic Characteristics		% (N)	% (N)
Gender *N* = 680	Male	59.5 (110)	40.5 (75)
	Female	64.0 (317)	36.0 (178)
Marital status *N* = 442	Married/DeFacto	70.3 (163)	29.7 (69)
	Never married	70.9 (139)	29.1 (57)
	Divorced/Widowed	78.6(11)	21.4(3)
Employment status *N* = 575	Employed	61.8 (136)	38.2 (84)
	Unemployed	68.8 (159)	31.2 (72)
	Not stated	67.7 (84)	32.3 (40)
State *N* = 680	Queensland	62.5 (80)	37.5 (48)
	Victoria	62.7 (168)	37.3 (100)
	Western Australia	66.7 (20)	33.3 (10)
	New South Wales	61.8 (136)	38.2 (84)
	South Australia	70.0 (14)	30.0 (6)
	Northern Territory	100.0 (3)	0.0 (0)
	Australian Capital Territory	100.0 (1)	0.0 (0)
	Tasmania	50.0 (5)	50.0 (5)
		Mean (standard deviation)	
Age at referral *N* = 680		55.5 (15.6)	52.1 (16.3)
SES (Decile^a^) *N* = 680		6.52 (2.9)	6.47 (2.8)
Clinical Characteristics		% (N)	% (N)
ICD-10 *N* = 493	Major depressive disorder	61.3 (100)	38.7 (63)
	Recurrent episodes of major depression	53.3 (32)	46.7 (28)
	Anxiety disorder	70.2 (66)	29.8 (28)
	Unspecified mental disorder	49.3 (71)	50.7 (73)
	Others (F34/F38/F40/F43/F45)	56.2 (18)	43.8 (14)
PHQ-9 Symptom Severity *N* = 675	No symptom (0–4)	71.4 (60)	28.6 (24)
	Mild symptom (5–9)	73.6 (120)	26.4 (43)
	Moderate symptom (10–14)	63.5 (115)	36.5 (66)
	Moderately severe (15–19)	54.0 (87)	46.0 (74)
	Severe (20–27)	52.3 (45)	47.7 (41)
GAD-7 Symptom Severity *N* = 670	None (0–4)	72.4 (89)	27.6 (34)
	Mild anxiety (5–10)	65.4 (157)	34.6 (83)
	Moderate anxiety (11–15)	60.7 (116)	39.3 (75)
	Severe anxiety (16–21)	56.0 (65)	44.0 (51)

^a^SES (Decile): Socio-economic status based on 2016 census data from Australian Bureau of Statistics (ABS), regions (post code) are given an Index of Relative Socioeconomic Advantage and Disadvantaged (IRSAD) score and assigned to a decile – with 10 representing the most advantaged region, one the most disadvantaged

Additionally, a total of 234 clients (30.9% of 757) had expressed suicidal ideation or risk symptoms^2^ at assessment. Of them, 22 (9.4%) were identified as ‘not suitable at assessment’; 34 (14.5%) either dropped out or declined further treatment; 44 (18.8%) were stepped up after one treatment session; 13 (5.6%) were stepped up after completing up to 6 treatment sessions; 90 (38.5%) completed the program while 31 (13.3%) remained ‘active cases’ at the time of reporting. There were no completed suicides during the study period.

#### Main analysis

Table 3 provides the effect size estimates Cohens *d* of pre-post change, recovery rates and reliable recovery rates with 95% CIs for PHQ-9 and GAD-7, including PP and ITT analyses. For those clients who adhered to the IAPT protocol, PP effect sizes were in the large range based on conventional standards (i.e., *d* = 0.2 small effect; *d* = 0.5 medium effect; *d* = 0.8 large effect [28]. The effect size of 1.03 for depression meant that, on average, those undergoing therapy would improve their score by + 1.03 standard deviation units at post-treatment. This is equivalent to moving from the 50th percentile to the 85th percentile in terms of improvement (reduction) in PHQ scores [29]. For anxiety, an improvement of + 0.99 standard deviations was comparable to a client moving from the 50th percentile to the 84th percentile following therapy. The recovery rate of 66% (95% CI: 61–72%) indicated that this proportion of clients moved from caseness at pre-treatment to not being at caseness post-treatment on both anxiety and depression measures. The reliable recovery rate of 62% (57–68%) was a combination of reliable improvement and recovery– the proportion of clients who showed both a change from caseness to not being caseness during the course of therapy and who also showed a reliable improvement in their score(s).

**Table 3:** Pre-post estimates for symptoms of depression (PHQ-9) and anxiety (GAD-7)

Analytic strategy	N	ES(PHQ)	ES(GAD)	N clinical cases	Recovery rate	Reliable recovery
PP	427	1.03(0.92–1.16)	0.99(0.88–1.11)	301	0.66(0.61–0.72)	0.62(0.57–0.68)
mITT^a^	584	0.88(0.79-0.98)	0.82(0.71–0.90)	410	0.60(0.55–0.65)	0.56(0.51–0.61)
ITT						
MI	680	0.78(0.69–0.86)	0.76(0.67–0.84)	497	0.53(0.48–0.57)	0.49(0.45–0.54)
LOCF	675	0.77(0.68–0.85)	0.75 (0.66–0.83)	497	0.51 (0.46–0.55)	0.47 (0.43–0.51)

*Abbreviations:* *ES* Effect size, *PHQ-9* Patient health questionnaire-9, Generalised Anxiety Disorder-7 questionnaire, *PP* Per protocol analysis, *(m) ITT* (modified) intent-to-treat analysis, *MI* Multiple imputation, *LOCF* Last observation carried forward

^a^Excluding stepped-up clients (*n* = 96)

The mITT analysis included all clients except those who stepped-up in therapy. Similar to PP results, effect sizes were in the large range and indicated client PHQ-9 scores improved by + 0.88 standard deviation units (50th percentile → 81st percentile) at post-treatment. GAD-7 scores improved by + 0.82 standard deviation units (50th percentile → 79th percentile). The recovery rate was 60% (95% CI: 55–65%) and reliable recovery, 56% (51–61%). For ITT (i.e., all clients included in the analysis) using multiply imputed datasets, the PHQ-9 effect size estimate of 0.78 (50th percentile → 78th percentile) was in the moderate range as was GAD-7 (0.76; 50th percentile → 78th percentile). The recovery rate was 53% (48–57%) and reliable recovery 49% (45–54%). Overall, there was an expected decrease in effect size, recovery rate and reliable recovery rate from PP analysis to the more conservative ITT analysis using MI method. The LOCF method provides the most (anti) conservative estimates but should be interpreted with caution as they do not account for error in imputed values.

Table 4 details results for sensitivity analysis performed via a pattern mixture approach with multiple imputation. Using the distributions *N*(M = 5.0, SD = 5.0) for PHQ-9 and *N*(M = 5.0, SD = 5.0) for GAD-7 to summarise the assumed difference between MNAR and MAR, there were no distinct changes between these estimates and MAR mechanism of missingness. Similar patterns of findings were found when further increasing the mean response above that predicted under MAR. In summary, these sensitivity analyses suggest that findings from MI estimates of recovery rates for PHQ-9 and GAD-7 were plausible under MAR assumption.

**Table 4:** Estimated effect of IAPT intervention from regression, multiple imputation under MAR, and multiple imputation under MNAR

Analysis	PHQ-9		*p*		GAD-7		*p*
	Estimate	95% CI			Estimate	95% CI	
LR	0.59	0.52–0.66	< 0.001		0.50	0.43–0.56	< 0.001
MI (MAR)	0.59	0.52–0.66	< 0.001		0.50	0.43–0.56	< 0.001
MI (MNAR)							
*N*(5, 5)	0.59	0.52–0.67	< 0.001	*N*(4, 4)	0.50	0.43–0.57	< 0.001
*N*(10, 5)	0.60	0.52–0.67	< 0.001	*N*(8, 4)	0.51	0.44–0.58	< 0.001
*N*(10, 20)	0.60	0.50–0.69	< 0.001	*N*(8, 16)	0.51	0.40–0.61	< 0.001

*Abbreviations:* *PHQ-9* Patient health questionnaire-9, Generalised Anxiety Disorder-7 questionnaire, *CI* Confidence interval, *LR* Linear regression, *MI* Multiple imputation, *MAR* Missing at random, *MNAR* Missing not at random

#### Secondary exploratory analyses

##### Symptom severity and treatment outcome

The recovery status and its relationship with clients’ initial symptom severity [18] was also analysed for those who completed treatment (N = 427). Pearson’s chi-square test showed a significant association between PHQ-9 symptom severity and associated recovery status with a small effect size for those who were at initial caseness (*N* = 247). Clients with ‘moderate symptom’ (10–14) and ‘moderately severe symptom’ (15–19) at baseline PHQ-9 had the highest recovery numbers. Similarly, for GAD-7 (*N* = 249), clients with ‘mild symptom’ (8–10) and ‘moderate symptom’ (11–15) had the highest recovery numbers (Table 5).

**Table 5:** Clients’ recovery status as per their symptom severity

	Recovered % (N)	Did not recover % (N)	Statistical analyses
PHQ-9 Symptom severity			
Moderate (10–14)	80.8 (93)	19.1 (22)	χ2 = 9.77, Cramer’s V = 0. 19, *p* = 0.008
Moderately severe (15–19)	67.8 (59)	32.1 (28)	
Severe (20–27)	57.7 (26)	42.2 (19)	
Total	72.0 (178)	27.9 (69)	
GAD-7 Symptom severity			
Mild (8–10)	80.8 (55)	19.1 (13)	χ2 = 7.92, Cramer’s V = 0.18, *p* = 0.019
Moderate (11–15)	68.1 (79)	31.8 (37)	
Severe (16–21)	58.4 (38)	41.5 (27)	
Total	69.0 (172)	30.9 (77)	

**Fig. 3:**
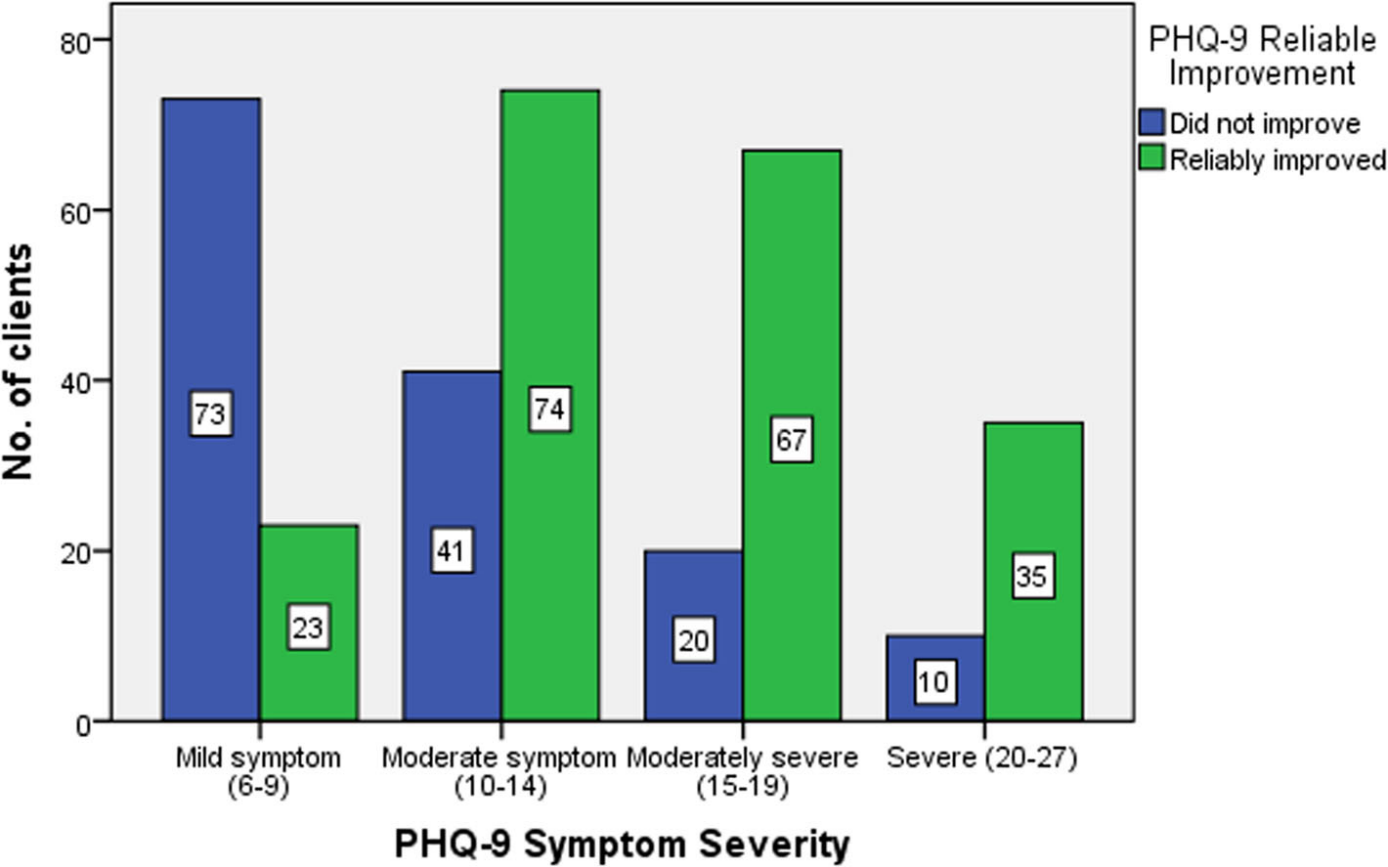
Association between PHQ-9 (Depression) relaible improvement and symptom severity for those who met the improvement criteria (PHQ-9 ≥ 6)

Of the 427 clients who completed the program, 282 (66.04%) also showed ‘reliable improvement’, 133 (31.15%) showed ‘no change’ and only 12 (2.81%) showed ‘reliable deterioration’ (Additional file 2). Pearson’s chi-square test showed that there was a strong association between clients’ PHQ-9 symptom severity and reliable improvement in their scores (*N* = 343, χ^2^ = 67.71, Cramer’s V = 0.44, *p* < 0.001). Clients assessed with ‘moderate’ (64.3%,*N* = 74), ‘moderately severe’ (77.0%,N = 67) and ‘severe’ depression symptoms (77.8%,*N* = 35) showed the most reduction in their PHQ-9 scores (Fig. 3).

**Fig. 4:**
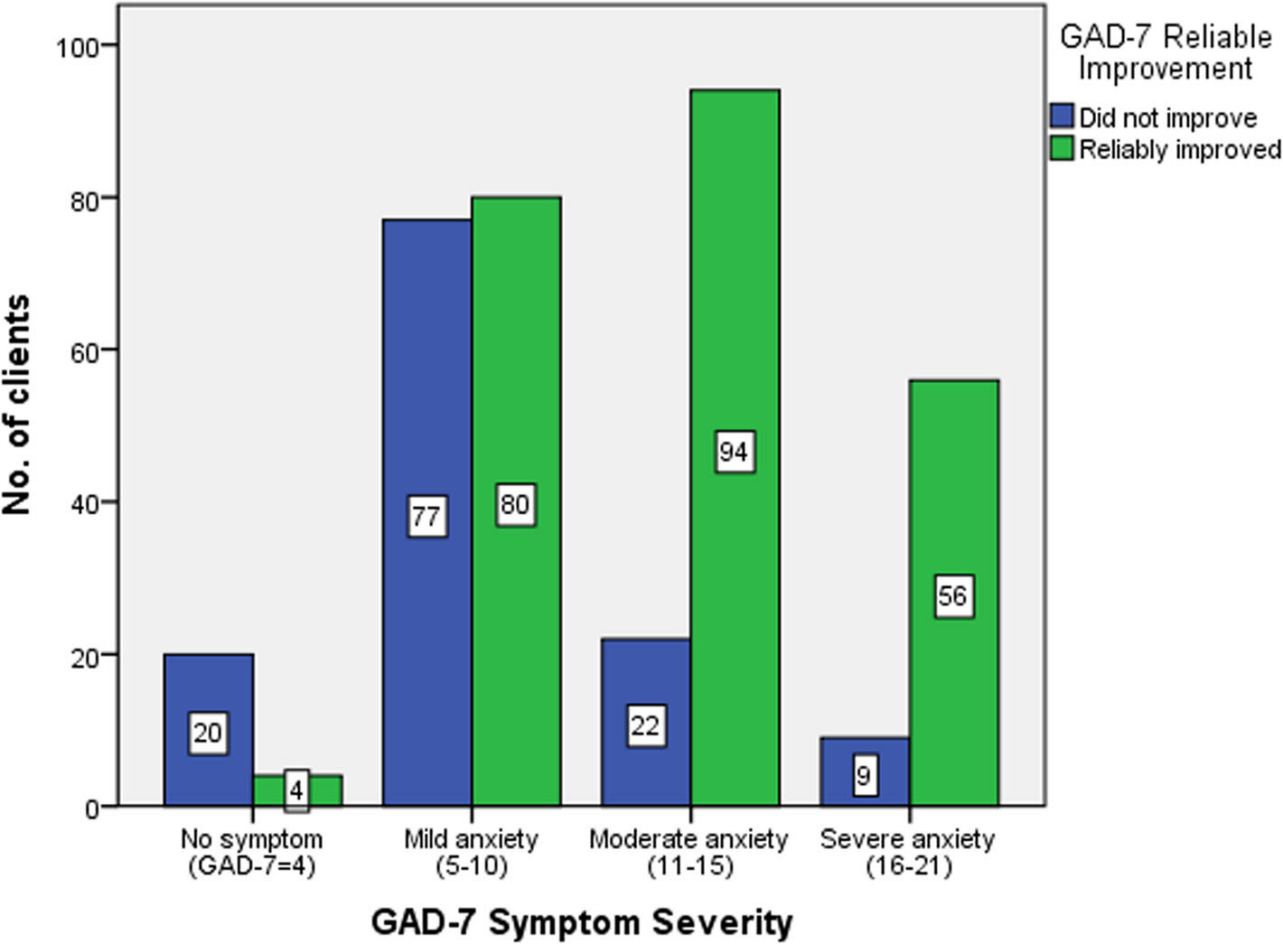
Association between GAD-7 (Anxiety) relaible improvement and symptom severity for those who met the improvement criteria (GAD-7 ≥ 4)

Similarly, the test showed that there was a fairly strong association between clients’ GAD-7 symptom severity and their reliable improvement (*N* = 362, χ^2^ = 63.83, Cramer’s V = 0.42, *p* < 0.001). Clients assessed with ‘moderate anxiety’ (81.0%,*N* = 94) and ‘severe anxiety’ (86.2%,*N* = 56) showed the most reduction in their GAD-7 scores (Fig. 4).

##### Treatment sessions

Of 427 clients who completed the treatment, the mean number of sessions completed was 5.0 (SD = 1.4) across an average duration of 10.5 weeks (SD = 10.6) with interquartile range (IQR), 1.14–15.7. Also, 62.9% (*N* = 300) completed all six treatment sessions.

Results showed that more clients who completed six sessions, reached the recovery threshold compared to those who had two to five treatment sessions (133 vs 67, Z = 6.6, *p* < 0.001). Results were similar for those who reliably improved (186 vs 96, Z = 7.57, *p* < 0.001).

##### Follow-ups

Of 427 clients who completed treatment, 282 (66.0%) also received 1, 3 or 6-months follow-up calls. Paired samples T-test [30] showed significant difference between the pre intervention and post follow-up PHQ-9 scores (pre-score = 10.91, post-score = 4.32, mean difference = 6.59, *p* < 0.001) and GAD-7 scores (pre-score = 9.14, post-score = 3.68, mean difference = 5.46, *p* < 0.001).

##### Coach variance

**Fig. 5:**
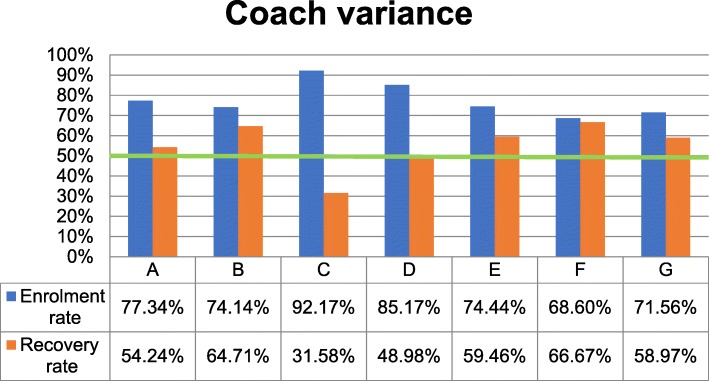
Coaches’ variances in enrolment and recovery rate

There were seven coaches between March’16 and July’17 in MindStep™. Fishers’ exact test [21] showed that coach C had the highest enrolment rate (92.2%, *p* = 0.001) but, interestingly, the lowest recovery rate (31.6%, *p* = 0.003) (Fig. 5). There was no significant difference between other coaches’ performance.

The test also showed that there was no significant difference among the coaches for number of clients who reliably improved, were stepped-up or followed up (*p* > 0.05) while taken into account their symptom severity.

However, GLM [26] showed that there was a significant difference among the coaches regarding number of treatment sessions, while controlling for clients’ symptom severity, age and gender (F = 4.79, *p* < 0.001). For all coaches, minimum and maximum number of sessions ranged between 2 and 8, with coach E having a significantly higher mean number of sessions (*N* = 6) than coach A (*N* = 5, p = 0.001), C (N = 5, *p* = 0.002) and D (N = 5, *p* = 0.011). Here, it is notable that clients of these later three coaches also had lower recovery rate than clients of coach E, but there was no statistical significant difference (*p* > 0.05).

#### Qualitative findings

Alongside quantitative methods, this study also employed qualitative methods to understand the acceptance and feasibility of the program. Twenty-one out of 70 randomly selected clients (including dropouts) consented to being interviewed for this part of the study. However, seven could not be contacted on the day of interviews and thus, a total of 14 clients were interviewed. Four of the seven coaches were interviewed. At the time of conducting the interviews, there were four active coaches who were employed from the start of the service, and they all consented to take part in interviews. The remaining three coaches started only 3 months prior to the evaluation and were not approached. Similar to preceding results, qualitative data also showed positive outcomes of the program.

##### Treatment satisfaction

**Fig. 6:**
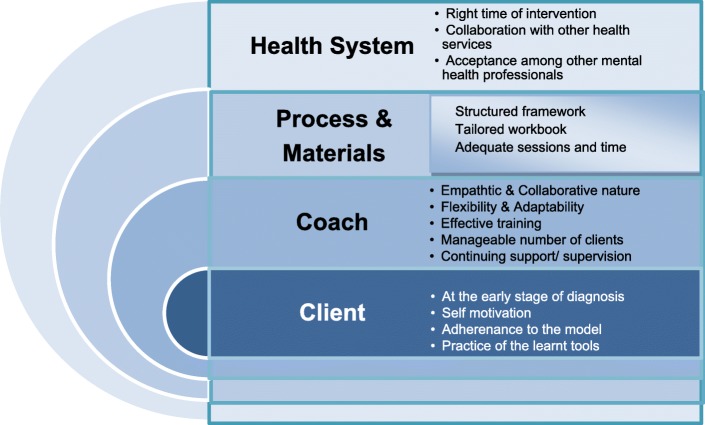
Key factors of a successful program

Based on the analysis of transcripts of clients’ and coaches’ interviews, key themes were identified and summarised in Fig. 6. The findings indicate that clients’ motivation and adherence, coaches’ empathetic and structured yet flexible coaching style, use of appropriate process and materials, and finally, collaboration with the existing health system to be able to intervene at the ‘right time’- all played important roles in the perceived success of MindStep™.

##### Program effectiveness

Clients and coaches were consistent in being positive about MindStep™, in particular how it is perceived amongst users and other health professionals. Both clients and coaches reported that symptoms of depression and anxiety had been appropriately diagnosed and managed.

*I just found MindStep actually much more valuable sometimes, and I just thought if only I had done this first, four years ago, instead of going through all the other stuff, and dealing through traumas, I honestly believe that I would have had a different experience in my recovery. (Client 5)*

*Well I think the strengths (of the program) are it really is working. We’re hearing it with our clients. People are finding that it’s helping them so that’s pretty amazing and I’m sure that comes from the fact that it is structured and firmly within a specific scope. (Coach D)*

##### Perception of coaching quality

All clients (including those who dropped out) thought highly of coaches’ professionalism and competency. They appreciated the empathy and the ability to build rapport by coaches.

*She was very professional. We made a time and she’d ring on time and it was very well run. I can’t say enough about that…I had a tendency just to go off track and she was very good, she always pulled me back in and was obviously trained well. (Client 4)*

*It always felt like he was there for me, not just for getting his numbers, like it didn’t seem like it was just his job, it seemed like he cared as well. (Client 6)*

##### Access

Clients valued the ease of access (intervention via telephone), materials (self-help guides, workbooks, pre-treatment diary), and delivery technique (shared-decision making process) of MindStep™.

*I found it easier over the phone in my own time, in my own environment. It was a bit more relaxing that way. (Client 2)*

*I think the thing that has helped quite a lot were the two workbooks that I went through and that sort of showed me the larger body of work that makes it all hang together…Also, knowing that I’ve got those at my fingertips. I know where to look in my own time. (Client 7)*

*Yeah, sort of being in control and being able to work together in setting the program whereas I think some people might feel face-to-face the counsellor/psychiatrist are running the show so to speak. This way you got to be a part of your own plan. (Client 9)*

##### Structure

Coaches valued the structured framework of delivery. They mentioned clients appreciated new ideas about how to manage symptoms and how the structure increased their faith in the MindStep™ and the coaches.

*…things like the maintenance cycle and the rationale which we do right at the beginning and the problem statement is often such a-they go like oh yes, you really heard me and that is my problem and yes that treatment-that would be amazing…It’s probably that trust and-it’s not rapport but you’ve sort of proven your worth quite early on which is a good engagement piece for the rest of the program. (Coach A)*

Coaches perceived that when clients attended regularly, completed their homework and practiced the skills, they ‘reaped the rewards’. Coaches also found the ability to tailor their delivery techniques to client needs helpful.

*…within that (structured framework) you still have the possibility to bring a bit of tailoring and individual personality and rapport building and things like the goal setting and getting to know your client that’s what makes it enjoyable and rewarding both as a coach and for the client I’d imagine. (Coach B)*

#### Barriers

Both clients and coaches commented that at times they struggled with completing the clinical measures (PHQ-9, GAD-7, WSAS etc.). Clients in particular felt there were ‘too many’ questions and they were ‘repetitive’. One client also felt the measures were not a true reflection of her fluctuating mental state.

*…for me it (scores) can change so quickly from moment to moment almost, that how I felt an hour ago might be completely different to how I feel now. (Client 14)*

*Doing the measures each week-I haven’t actually heard from the clients too much but that’s annoying...some mention it. I think maybe as a coach you’re doing them every day, five or ten times, and it’s just painful. (Coach C)*

Coaches also reported that the process of completing homework, keeping a diary etc. can be overwhelming for some clients.

One client who dropped out felt that MindStep™ might be more suited to a ‘first timer’.

*I’ve been in and out of hospital and seen counsellors for quite a long time and I felt like the content wasn’t new, that I’d heard it before…If it was someone who is experiencing depression or anxiety as a result of perhaps a family death or one thing…that perhaps it might have been more beneficial for those groups. (Client 14)*

##### Sustainability

Clients were confident in the program’s sustainability. However, coaches were less sure about how clients would cope during a crisis on their own after MindStep™ was completed.

*The people who are going well, some of them are honestly so confident with what they’ve learnt that I do feel confident for them. But there’s also a big chunk who should a situation arise that’s particularly stressful or sad as it would be for anyone I don’t know if they’ve got the resilience there simply because of the chronic nature of what they’ve been through. (Coach A)*

*I’m introducing that (relapse prevention) at say session 6 or session 7-which is fine but you kind of want to really embed it and I do wonder for some of my clients whether they’re going to be able to pick that up and run with it. (Coach C)*

One coach suggested adding ‘crisis intervention’ planning after initial sessions to help cope and respond to future crisis. Additionally, another suggested better collaboration with traditional mental health service providers can enhance the acceptability and effectiveness of MindStep™.

## Discussion

Unlike any other existing LiCBT services that are based on IAPT, the MindStep™ program is novel because it has been developed within the private health insurance space, is completely telephone-based, and serves the needs of people with not only mild and moderate symptom, but also more severe depression and anxiety. It also receives the vast majority of its referrals via private health insurers for clients identified following hospital discharge.

### Key results

The positive findings replicate recovery rates and clinical outcomes delivered in other settings, and add substantially to the body of knowledge accumulating for LiCBT and its application in Australia’s mental health stepped model of care. The findings confirm Mindstep™’s feasibility and acceptability in the Australian private health system, and that it can achieve benchmark recovery rates of > 50% in people with recent mental health hospital admission [18]. Our results are also mostly comparable to the recent Norwegian report [17], using both complete case and ITT analytic strategies.

Mindstep™ clients’ symptom severity at enrolment is comparable to the previously reported in the literature [12, 16, 18], and MindStep™ was also able to achieve comparable recovery rates of > 50% once the program was fully established. Qualitative findings confirmed the acceptance of telephone-based delivery of LiCBT by participants and the use MindStep™ coaches who received accredited mental health training, but did not come from a traditional mental health service background. In particular, this approach may help to address the ‘tyranny of distance’, including problems with access to services and resourcing issues often experienced within Australia’s vast rural and remote regions.

Overall, the results showed that clients with ‘moderate’ and ‘moderately severe’ depression (10–19 on PHQ-9) or ‘mild’ and ‘moderate’ anxiety (8–15 on GAD-7) were more likely to reach the threshold of ‘recovery’. Also, clients who had ‘moderately severe’ and ‘severe’ depression (15–27 on PHQ-9) showed the highest ‘reliable improvement’ in their PHQ-9 scores. Similarly, clients with ‘moderate’ and ‘severe’ anxiety (11–21 on GAD-7) showed the highest ‘reliable improvement’ in their GAD-7 scores. The results showed that MindStep™ could result in clinically significant symptom reduction, whether clients reached the definition threshold for ‘recovery’ or not. Hence, measures of recovery and reliable improvement can both offer meaningful clinical outcomes to assess mental health interventions for depression and anxiety.

An international meta-analysis of 16 studies comparing low intensity interventions with usual care found that patients who had more severe symptoms of depression at baseline showed at least as much clinical benefit as those with less severe symptoms, within the stepped care model [31]. Further research is needed to understand the cost effectiveness, retention strategies and referral options of providing LiCBT to people at different symptom thresholds, with an aim to enable everyone who needs this evidence-based treatment to have access at a time and place that is convenient to them [32–34].

MindStep™ can be delivered to large numbers of people across geographical boundaries for people with symptoms of depression and anxiety. It could continue to explore options for serving those clients with more severe needs; including veterans and others with potentially more complex presentations such as Post Traumatic Stress Disorder (PTSD), chronic pain and post-natal depression. It also has the potential to address clients’ comorbid mental and physical conditions [33]. This would have implications for coaches’ skillsets, levels of support and supervision, clinical governance and management of risk, interactions with other programs, and the overall development of the clinical model.

Compared to the UK IAPT post-assessment dropout rate benchmark of 35% [18], MindStep™ showed much lower dropout rate (12.8%). Of interest, White [34] reported that globally, clients using a primary care clinical psychology service are more likely to cancel or not attend their first appointments (17–84%). Thus, dropout between referral and assessment and soon after assessment, are a well-known phenomenon within the delivery of psychological therapies [35, 36]. Also, of these client cohorts, only 17–65% seem to complete all treatment sessions, which is comparable to MindStep™ completion rate of 62.9%.

Of the other variables investigated, this analysis showed that coaches who provided five sessions or more of LiCBT, achieved higher client recovery rates. Therefore, clients should be encouraged to complete a minimum of five sessions. Battersby et al. [37] found that improved outcomes from this sort of problem-solving therapy were associated with an increased number of sessions. However, there was no significant difference between coaches’ performances based on clients’ reliable improvement, step-up or follow-up numbers when controlling for clients’ symptom severity. Except for one coach, there was also no significant difference in their clients’ enrolment and recovery rate. To enhance the role of coaches and to ensure effective and consistent coaching to address variations across coaches and within individual coaches, more shadowing, mentoring and transition steps to accommodate the learning and support needs of new coaches could be employed in the future.

The qualitative findings showed that the coaches were keys to the success of the program. The coaches’ interviews revealed that even though the model itself was well structured and formulated, it also required some personalisation to client need. If a client sticks with the plan and cooperates with the coach, he or she is likely to get a more positive outcome. However, even when the program finishes, the client would require practicing their newly learnt tool of ‘self-management’ to keep them moving forward. This suggests a clear need to improve transfer and communication with the person’s primary healthcare provider or others who may be involved in supporting their ongoing mental health and wellbeing.

Thus, the results of this study suggest a number of issues for improvement of the MindStep™ program. Firstly, the program should continue to target clients across the existing range of severity of conditions and explore options for clearly defined clinical pathways for those with more severe needs. MindStep™ should increase accessibility to men clients as well. It should also and consider expanding to include after-hours delivery of service to some clients. The program should be promoted on a larger scale to help mental health professionals to better understand the program. In particular, this should include how the program is complementary to traditional services, not conflicting or competing with these services. Because it is a totally phone-based service, MindStep™ can reach a large number of the target population, especially those living in remote areas. A Dutch qualitative study using Normalisation Process Theory to investigate the implementation of stepped care in primary care found that primary care clinicians’ increased awareness and understanding of stepped care (inclusive of LiCBT services) improved their ability to differential patient groups, target antidepressant prescribing more effectively, and have better working relationships with patients and colleagues. A range of barriers were also identified, suggesting areas for more intensive focus and support, such as addressing attitudes, service culture, competition between disciplines, and poor organisational infrastructure that hampered effective communication [38, 39].

For clients, the service could consider how it can be tailored to accommodate the needs of clients with lower literacy levels, inclusive of coach training to support more flexible delivery of the program to these clients. During the pilot phase, LiCBT workbooks developed by Exeter University in the UK were used to deliver the service, with cases and support information tailored to the UK context. This is not ideal and, therefore, Australian versions of the workbooks have been developed by Flinders University. Each of these workbooks, and the sessions in which they are used by coaches and clients, have a clear focus on crisis intervention and relapse prevention as part of closure and transfer work with clients. The next step should be to support online availability of the workbooks to enable their completion electronically by clients.

### Strengths and limitations

A key strength of this study is that the findings provide essential precursors to high quality clinical trials - a much needed step to developing the trajectory of IAPT research in the Australian context. On this note, we presented ITT findings as secondary data due to the observational nature of our study design. A future RCT would afford more robust data concerning therapy effectiveness from an ITT perspective.

A limitation was the use of the single imputation method LOCF. This approach misses the informative properties of missingness and does not account for error in imputed values. However, we included LOCF findings to provide an opportunity for comparison with other missing data techniques- namely multiple imputation and complete case analysis. Our findings suggested that LOCF estimates were indeed anti-conservative by introducing bias from *“worse than true (but missing)”* values [40].

One of the study’s other inherent limitations was the absence of a control group. A further limitation was the use of the self-reported PHQ-9 and GAD-7 as key measures for determining clinical levels of depression (PHQ-9 > 9) and anxiety (GAD-7 > 7). Although they have good psychometric properties, Richards & Borglin [30] remind us that they do not formally diagnose patients. Rodgers et al’s [41] systematic review recommended that future studies should include “participants in remission or recovery from depression, and evaluate the quality of the intervention and consistency of delivery across practitioners where appropriate. The occurrence of relapse or recurrence should be measured using established methods” (p.1). For our study, diagnoses (ICD-10) were available only at baseline from the external referral sources (hospitals etc.) and were not routinely collected as part of the IAPT protocol. Further longitudinal study is also required to determine the program’s potential to reduce hospital readmissions and associated costs.

Also, this evaluation did not consider the needs of clients from non-English speaking backgrounds or Indigenous populations. Further adaptation of the processes and materials used in the service, and their evaluation, is needed. More generally, the potential impact of client literacy levels was not included in this analysis, even though it is known to be important for promoting access to this type of service [6]. Further research is also needed to understand coach qualities and actions that may influence client outcomes. Gellatly et al. [14] have indicated that little is known about these aspects of LiCBT and what role non-specific coach factors might play, though qualifications and background of coaches appears to make little difference to client outcomes. Further research is also needed on whether the mode of delivery of LiCBT matters to client outcomes.

## Conclusions

MindStep™ appears to be a promising telephone-based LiCBT intervention for people who have had a recent hospital admission for anxiety and/or depression. The results of this evaluation are encouraging and suggest that telephone-based LiCBT is acceptable, feasible and effective at addressing the mental health needs of people to bridge a gap in the transition from acute to community based mental health care. It appears to add an important component within the suite of options of Australia’s stepped approach to mental health care; therefore, replication and further evaluation, including more longitudinal follow-up data to investigate to the stability of improvements, is warranted. This type of service is also important for Australia because of its geography as a large country with several rural and remote communities that are underserved by the healthcare system, and that have poorer health outcomes as a consequence of that geography.

## Additional files

Additional file 1: MindStep^TM^ [42–45]. (DOCX 17 kb)

Additional file 2: Overall Reliable Improvement. (DOCX 15 kb)

## Abbreviations

AU: Australian Unity
GAD-7: Generalized Anxiety Disorder
IAPT: Improving Access to Psychological Therapy
ICD-10: International Classification of Diseases
LiCBT: Low intensity Cognitive Behavioural Therapy
NICE: The National Institute for Health And Care Excellence
PHQ-9: Patient Health Questionnaire
PTSD: Post Traumatic Stress Disorder
SD: Standard deviation
SPSS: Statistical Package for the Social Sciences
WSAS: Work and Social Adjustment scale

## Footnotes

1 MindStep™ was developed by Remedy Healthcare, a wholly owned subsidiary of Australian Unity. Remedy is a leading provider of highly targeted, evidence-based health coaching programs and out of hospital care for people with long term conditions to support their capacity for self-management. These programs have been funded by private health insurers, employers, aged care providers and other corporate health organisations since 2008.

2 Suicide symptoms were determined by the PHQ-9 last question that asked ‘thoughts that you would be better off dead or hurting yourself in some way- not at all=0, several days=1, more than half the days=2, nearly every day=3’. Clients with a score higher than 0 are reported here.
