# Supplementary material for: Outcomes of telephone-delivered low-intensity cognitive behaviour therapy (LiCBT) to community dwelling Australians with a recent hospital admission due to depression or anxiety: MindStep™
Source: BMC Psychiatry. 2019 Jan 3;19:2. doi: 10.1186/s12888-018-1987-1 (PMC6319009; doi:10.1186/s12888-018-1987-1)
Supplement: Supplementary file 1 — MindStepTM [42–45]. (DOCX 17 kb) [file 12888_2018_1987_MOESM1_ESM.docx]

Additional file 1- ***MindStep^TM^***

MindStep^TM^ forms part of the stepped care model to address the transition from acute to community mental health care in Australia. It can be accessed from anywhere in Australia. Thus, it aims to bridge the care provided by mental health specialist and the general practitioner (GP), providing evidence-based low intensity guided cognitive behaviour therapy (LiCBT) for people recently discharged from hospital with a diagnosis of depression and/or anxiety.

This LiCBT model is delivered by mental health coaches trained by the Discipline of Psychiatry at Flinders University in South Australia in a postgraduate course. Coaches are selected on the basis of their communication, listening and empathy skills, rather than having a formal mental health or psychology qualification. MindStep^TM^ had seven coaches during the study period. Only one coach is a qualified mental health professional; whereas, others have health or community worker qualifications, but they are not qualified clinical health professionals. However, they are trained and closely supervised by Flinders University mental health professionals who have postgraduate qualifications in CBT. Selected coaches receive training that is conducted over 12-months using curriculum and competency standards that are aligned with the UK IAPT services. The curriculum includes evidence-based brief guided self-help using cognitive behavioural skills, complemented by training to support social prescribing and signposting to community services.

Coaches conduct symptom assessments using *Patient Health Questionnaire* (PHQ-9) [18] and *Generalized Anxiety Disorder* (GAD-7) [19] at each contact and the data is entered into the software system in real time. Through automated alerts triggered by clinical thresholds, the system allows supervisors to have full visibility of client’s progress in real time and communicate quickly if there is a need to step up care or to refer to higher intensity treatments for failure to progress, deterioration or high risk [20]. Additionally, the Work and Social Adjustment scale (WSAS) is used at each encounter to assess level of disability associated with symptoms [21].

Thus, MindStep^TM^ model allows easy access, rapid assessment, flexible telephone delivery, outcomes focus with data collection at every session, and real-time supervision using an online data sharing platform. It is designed to reach high volumes of people across geographical boundaries, including clients from rural and remote regions, who may not otherwise have access to evidence based therapies.

#### Safety and ethical considerations

Ethics approval was gained from the Flinders University Social and Behavioural Research Ethics Committee, with an amendment sought to conduct this further evaluation (project no. 7010). Clients assessed at high risk (with clear clinical thresholds and monitored in real time by clinical supervisors at Flinders University) at any point were immediately ‘stepped-up’ to their usual mental health provider or community mental health or crisis support, depending on the urgency and existing support networks. Clear protocols were in place and tested regularly to ensure staff compliance; including currency of telephone and crisis contacts.

The online clinical software system enables all clinical data to be viewed and reviewed in real time by coach and clinical supervisor, with structured triggers to alert the supervisor, if required. This protocol-driven supervision model ensured high risk or complex symptoms were identified immediately and coaches could step-up care to a higher intensity therapist such as a GP, psychologist, psychiatrist or community emergency team if suicide risk or harm to others was identified.

MindStep^TM^ is a significant development because it is the first adaptation of IAPT in Australia to target people from participating private health insurance funds with a recent hospital admission due to depression and/or anxiety. If successful, this novel approach can add significantly to bridge identified mental health service gaps for people transitioning from acute to the community care settings.
