# Supplementary material for: Outcomes of telephone-delivered low-intensity cognitive behaviour therapy (LiCBT) to community dwelling Australians with a recent hospital admission due to depression or anxiety: MindStep™
Source: BMC Psychiatry. 2019 Jan 3;19:2. doi: 10.1186/s12888-018-1987-1 (PMC6319009; doi:10.1186/s12888-018-1987-1)
Supplement: Supplementary file 2 — Overall Reliable Improvement. (DOCX 15 kb) [file 12888_2018_1987_MOESM2_ESM.docx]

Additional file 2

Overall Reliable Improvement

| **Score Change** | **Parameters** | **Numbers** | | **Rate** | |
| --- | --- | --- | --- | --- | --- |
| Improvement | Reliable reduction^a^ PHQ-9 & reliable reduction GAD-7 | 150 | | 35.12% | |
|  | Reliable reduction PHQ-9 & no reliable reduction GAD-7 | 48 | | 11.24% | |
|  | No reliable reduction PHQ-9 & reliable reduction GAD-7 | 84 | | 19.67% | |
| No Change | No reliable change^b^ PHQ-9 & no reliable change GAD-7 | 132 | | 30.91% | |
|  | Reliable reduction PHQ-9 & reliable increase GAD-7 | 1 | | 0.23% | |
|  | Reliable increase PHQ-9 & reliable reduction GAD-7 | 0 | | 0% | |
| Deterioration | Reliable increase^c^ PHQ-9 & reliable increase GAD-7 | 3 | | 0.70% | |
|  | Reliable increase PHQ-9 & no reliable change GAD-7 | 5 | | 1.17% | |
|  | No reliable change PHQ-9 & reliable increase GAD-7 | 4 | | 0.94% | |
| **Sum of all clients included in this analysis** | | | | | |
| Sum of clients meeting ‘improvement’ criteria | | | 282 | | 66.04% |
| Sum of clients meeting ‘no change’ criteria | | | 133 | | 31.15% |
| Sum of clients meeting ‘deterioration’ criteria | | | 12 | | 2.81% |
| **Total** | | | **427** | | **100%** |

a. Reliable reduction: If a client’s score reduces by 6 points or more on PHQ-9 and 4 or more on GAD-7

b. No reliable change: If a client’s score change is between -5 and 5 on PHQ-9 and -3 and 3 on GAD-7

c. Reliable increase: If a client’s score increases by 6 points or more on PHQ-9 and 4 or more on GAD-7
